# Developmental Effects of Exposures to Environmental Factors: The Polish Mother and Child Cohort Study

**DOI:** 10.1155/2013/629716

**Published:** 2013-09-26

**Authors:** Kinga Polanska, Wojciech Hanke, Wojciech Sobala, Malgorzata Trzcinka-Ochocka, Danuta Ligocka, Slawomir Brzeznicki, Halina Strugala-Stawik, Per Magnus

**Affiliations:** ^1^Department of Environmental Epidemiology, Nofer Institute of Occupational Medicine, 8 Teresy Street, 91-348 Lodz, Poland; ^2^Department of Toxicology and Carcinogenesis, Nofer Institute of Occupational Medicine, 8 Teresy Street, 91-348 Lodz, Poland; ^3^Department of Chemical Safety, Nofer Institute of Occupational Medicine, 8 Teresy Street, 91-348 Lodz, Poland; ^4^The Foundation for Children from Copper Basin, 10 Okrzei Street, 59-220 Legnica, Poland; ^5^Division of Epidemiology, Norwegian Institute of Public Health, P.O. Box 4404 Nydalen, 0403 Oslo, Norway

## Abstract

This paper estimates the effects of exposure to environmental factors, including lead, mercury, environmental tobacco smoke (ETS), and polycyclic aromatic hydrocarbons (PAH), on child psychomotor development. The study population consists of mother-child pairs in the Polish Mother and Child Cohort Study. Prenatal and postnatal exposure to environmental factors was determined from biomarker measurements as follows: for lead exposure—cord blood lead level, for mercury—maternal hair mercury level, for ETS—cotinine level in saliva and urine, and for PAH—1-hydroxypyrene (1-HP) in urine. At the age of 12 (406 subjects) and 24 months (198 subjects) children were assessed using Bayley Scales of Infant and Toddler Development. There were no statistically significant effects of prenatal exposure to mercury or 1-HP on child psychomotor development. After adjusting for potential confounders, adverse effects of prenatal exposure to ETS on motor development (**β** = −2.6; *P* = 0.02) and postnatal exposure to ETS on cognitive (**β** = −0.2; *P* = 0.05) and motor functions (**β** = −0.5; *P* = 0.01) were found. The adverse effect of prenatal lead exposure on cognitive score was of borderline significance (**β** = −6.2; *P* = 0.06). The study underscores the importance of policies and public health interventions that aim to reduce prenatal and postnatal exposure to lead and ETS.

## 1. Introduction

At present, special attention is given to prenatal and childhood exposures to lifestyles and environmental factors and their impact on child neurodevelopment. Among a variety of contaminants, exposure to lead, mercury, and tobacco constituents have been widely investigated whereas the exposure to polycyclic aromatic hydrocarbons (PAH) has been less frequently examined.

The majority of studies have confirmed neurodevelopmental effects of blood lead levels (BLL) above 10 *μ*g/dL. This includes lowered intelligence, behavioral problems, deficits in academic achievements, and problem solving, as well as reductions in visual/spatial, motor, and language skills [1–3]. Thanks to public health and regulatory activities the BLL significantly decreased, however, still many studies have found adverse effects of levels below 10 *μ*g/dL. The pooled analysis of international studies, performed by Lanphear et al. [2], indicates that the estimated IQ point decrement associated with an increase in BLL from 2.4 to 10 *μ*g/dL was 3.9 units (95% CI 2.4–5.3). The decrease was 1.9 units when BLL increased from 10 to 20 *μ*g/dL (95% CI 1.2–2.6), while it was 1.1 units (95% CI 0.7–1.5) from 20 to 30 *μ*g/dL. This observation suggests that the rate of decline in IQ scores might be even larger at BLL below 10 *μ*g/dL. Together with other data it indicates that no level of lead exposure appears to be safe and even the current relatively low levels of exposure in children are associated with neurodevelopmental deficits [1]. 

The evidence for adverse effects of prenatal mercury exposure on child neurodevelopment is more inconclusive. Exposure to mercury in some studies has been associated with poorer psychomotor scores including cognitive, memory, and attention functioning as well as motor and language abilities and visual information processing [4–9], whereas no associations have been found in other studies [10–14]. 

The published data that has evaluated the impact of environmental PAH exposure, based on personal air sampling or measurements of PAH-DNA adducts levels, on child psychomotor development, have been derived from US, Chinese, and Polish cohorts. These studies indicate that prenatal PAH exposure is associated with developmental delay within the first years of life and reduced cognitive development and IQ at the age of 5 years [15–18]. In addition, higher levels of cord PAH-DNA adducts have been associated with reduced scores on neurocognitive and motor tests as well as behavioral problems, alone or in combination with ETS exposure [19–21]. 

The impact of pre/postnatal ETS exposure, as the result of active and passive maternal smoking during pregnancy and/or child's exposure to ETS from tobacco products smoked in their surroundings, has been widely investigated [22]. Some studies indicate that such exposure is harmful to the developing nervous system and established a link between ETS exposure and neurobehavioral abnormalities in both infants and children [22]. Other analyses have indicated that the association of ETS exposure to poorer psychomotor development is strongly confounded by parental characteristics or can be related to limitations in study design and exposure assessment [22]. 

The mostly pointed limitations of the studies focused on the impact of environmental factors on child neurodevelopment are related to the study design and analysis such as retrospective study design, exposure assessment based on questionnaire data, and lack of adequate control for confounding factors. The current study contributes to elimination of the above obstacles. Prospective cohort study design (REPRO_PL cohort) enables identification of variety of exposures that may influence children's neurodevelopment, verification of such exposures by biomarker measurements, and notification of any changes in exposure levels. In current study, the exposure to chemicals was based on measurement of biomarkers which allows for better assessment of exposure and its critical windows. Additionally, the inclusion of potential confounding factors and coexposures is the advantage of current analysis comparing to previous studies.

 The aim of this study was to estimate the association between exposure to environmental factors including lead, mercury, ETS, and PAH and child psychomotor development based on data from the prospective Polish Mother and Child Cohort Study (REPRO_PL). 

## 2. Materials and Methods

### 2.1. Study Design and Population

The Polish Mother and Child Cohort Study (REPRO_PL cohort) was established in 2007. The complete description of the methodological assumptions has been published elsewhere [23, 24]. Women were recruited during first trimester of pregnancy at maternity units or clinics in selected regions of Poland if they fulfilled the following inclusion criteria: single pregnancy up to 12 weeks of gestation, no assisted conception, no pregnancy complications, and no chronic diseases as specified in the study protocol. 

Women were interviewed 3 times during pregnancy (once in each trimester) to collect and update demographic and socioeconomic data, medical and reproductive history, information about occupational exposure, and lifestyle factors. At each visit biological samples were collected from the women (first visit: saliva and blood; second visit: saliva, urine, and blood; third visit: saliva, urine, blood, and hair). Additionally we collected umbilical cord blood and detailed information regarding pregnancy outcomes and children's health. 

One year after the child's birth an invitation letter was sent to mothers participating in the REPRO_PL cohort proposing to have the child's exposure, health status, and neurodevelopment examined. Within the next two weeks a phone call was made to schedule a (mother and child) visit at the medical center for an examination by a pediatrician as well as a psychologist/child development specialist. The same procedure was repeated when the child was 24 months old. 

Finally, 411 children were examined at one year of age (out of 565 invited children) and 198 children were examined again at the age of 2 years (out of 313 invited children). Ninety-eight children, who had been examined at around 12 months of age, have not yet been examined at 24 months as they have not reached this age. 

### 2.2. Assessment of Children's Prenatal and Postnatal Exposure to Environmental Factors

Lead exposure during the prenatal period was assessed based on cord blood lead level (BLL) (233 samples). Blood samples were collected using S-Monovette with lithium heparin as anticoagulant and frozen at −20°C until analysis. For the analysis, the inductively coupled plasma mass spectrometry (ICP-MS) (Perkin Elmer Elan-DRCe) was used. The laboratory at the Nofer Institute of Occupational Medicine (NIOM) that performed the analysis is accredited by the Polish Center of Accreditation (Certificate AB215) according to PN/EN ISO/IEC 17025 to perform the analysis of the level of lead and cadmium in the blood in the fields of occupational medicine and environmental health.

For the assessment of prenatal exposure to mercury, hair samples (clipped close to the scalp from the back of head) were collected from the women between the 30th and 34th week of pregnancy (227 samples). The hair samples were stored in a paper envelope at room temperature. Total mercury concentration in hair samples was analyzed at NIOM laboratory using cold vapour atomic absorption spectrophotometry (CVAAS) according to the method described elsewhere [25].

Prenatal exposure to tobacco constituents was assessed based on cotinine level in saliva collected from women 3 times during pregnancy (once in each trimester). Saliva samples collected in first trimester of pregnancy were available for 362 women, in second for 132, and in third for 345 women. For 14 women we did not have any saliva samples so they were excluded from the models assessing the impact of prenatal tobacco smoke exposure on child neurodevelopment. For valid prenatal tobacco smoke exposure assessment we have calculated cumulative value taking into account all available data on cotinine level for each women (all three measurements were available for 109, two for 228, and one for 55 women). If the woman does not indicate the changes in smoking status during pregnancy, the mean value from available measurements was calculated. If she declares quitting smoking following procedure was applied, first such information was verified by cotinine level in saliva and then the duration period for smoking and nonsmoking status were taken into account in cumulative value calculation. Child exposure to environmental tobacco smoke was assessed based on cotinine level in urine collected at each visit scheduled for child health and neurodevelopment assessment (228 samples collected at 12 months of age and 116 samples collected at 24 months of age). The cotinine level in saliva and urine was analyzed at NIOM using high performance liquid chromatography coupled with tandem mass spectrometry/positive electrospray ionisation (LC-ESI+MS/MS) and isotope dilution method. This procedure has been validated under ISO 17025 criteria and accredited by the Polish Center of Accreditation (Certificate AB215).

Assessment of prenatal exposure to PAH was based on measurement of 1-hydroxypyrene (1-HP) in urine collected in 20–24 weeks of pregnancy (384 samples) using high performance liquid chromatography (HPLC). The analysis was performed at NIOM laboratory based on the method described by Jongeneelen et al. [26]. 

### 2.3. Child Neurodevelopment Assessment

Children's neurodevelopment was assessed at around 12 months (range, 10 to 18 months) and repeated at around 24 months of age (range, 23 to 30 months) using Bayley Scales of Infant and Toddler Development (third edition—Bayley-III) [27]. The examination was performed at pediatric, allergy or diagnostic, prophylactic, and therapeutic centers at two University Hospitals in Lodz and at the Foundation for Children from Copper Basin in Legnica. The testing was done in the presence of the mother or relative by trained psychologist or child development specialist. All analysis were performed with the adjustment of examiner who performed the test. Bayley-III is an individually applied examination that assesses the developmental functioning of children up to 42 months. The test presents children with situations and tasks designed to produce an observable set of behavioral responses. It assesses five developmental areas: cognitive, motor, language, social-emotional, and adaptive behavior. In current analysis, we have focused on child cognitive, motor, and language development. The cognitive development was measured using age-appropriate activities such as puzzle completion, matching and recognizing pictures, and pretending to play. Motor scale evaluates fine motor skills, such as visual tracking, reaching and grasping, as well as gross motor skills, such as sitting, crawling, standing, jumping, and walking up and down the stairs. In the area of language, Bayley-III assesses the development of communication skills including recognizing and naming objects. The child psychomotor development measured by row score/chronologic age was yielded with each subtests, and composite scores for language, motor scales, and composite score equivalent for cognitive scale were generated based on such data. 

We excluded from the analysis data for 5 children whose Bayley tests were of poor quality because of pathologies or less-than-optimal cooperation of the child. Finally the analysis was performed for 406 one-year-old children and for 198 children at age of two years.

### 2.4. Statistical Analysis

Statistical inference was conducted in two stages. First, univariable analysis was performed using nonparametric tests of significance between the Bayley's test results and the potential confounding variables identified based on the literature review. Covariates evaluated were parental age, parental education, marital status, socioeconomic status, alcohol consumption during pregnancy, child gender, type of delivery, gestational age, biometric indicators at birth, breastfeeding, number of siblings, and nursery attendance. The following confounding variables for multivariable model were identified (*P* < 0.1): child gender, parental age, parental education, and for cognitive development additionally marital status and nursery attendance. Saliva cotinine and cord blood lead levels were log transformed. Evaluation of the impact between environmental biomarkers and children psychomotor performance was carried out according to the following procedure: first conducting a linear regression estimation with random effect together for children surveyed in the first and second year of life (604 records) and then if on the basis of the significance test (*P* ≤ 0.05) the need for a separate model for both groups of children was identified, simple regression model without random effect was made separately for one- and two-year-old groups. In first model confounding effects of the examiner who performed the test and the age of the child at examination were taken into account. If impact of biomarker is significant according to the statistical test (*P* < 0.05), then the second model including all potential confounders was also conducted. In the third model the additional confounders were added into the analysis as follows: (1) cotinine level during first years of life in the analysis of the impact of prenatal tobacco smoke exposure on child motor development, (2) cotinine level during pregnancy in the analysis of the impact of postnatal ETS exposure on child cognitive, language, and motor development, (3) cotinine level during pregnancy and within first years of life in the analysis of the impact of lead exposure on child cognitive development, and (4) cord blood lead level in the analysis of the impact of postnatal ETS exposure on child cognitive development. Statistical reasoning were conducted based on statistical tests with a significance level of 0.05. The analysis was performed using the R package.

Finally, based on availability of data of biomarker level, psychomotor development test results and confounding variables, the model assessing the impact of environmental exposure on outcomes of interest (with the data combined for children examined in the first and second year of life) was performed for 326 records for lead, 303 records for mercury, 590 records for prenatal, and 333 for postnatal ETS and 576 records for PAH exposure evaluation. 

## 3. Results

### 3.1. Child and Parental Characteristics

Demographic and exposure characteristics of mothers and their children are summarized in Table 1. Data are presented for the 406 subjects as the basic group for analysis. There were no statistically significant differences regarding demographic, socioeconomic, and newborn parametric data between one- (406 subjects) and two- (198) year-old groups. 

About 53% of children were girls. On average the children were born at 39 weeks of gestation (±1.4 week) with the mean birth weight of 3353 g (±488 g) and length of 55 cm (±2.9 cm). The mean newborn head circumference was 34 cm (±1.6 cm) and chest circumference 33 cm (±1.8 cm). About 45% of the children were breastfeed at least 6 months after birth or longer. Nursery attendance was indicated for 7% of one-year-old children and 19% at the age of two. Fifty-eight percent of the children did not have the siblings. The mean age of the children at Bayley test administration was 12.4 months (±1.3 months) and for repeated examination 24.4 months (±1.7 months). The mean maternal age was 30 (±4.4 years) years and paternal age 32 years (±5.5 years). Most of the women (59%) had a university degree. This level of education was indicated by 40% of fathers. Also most of the women were married (77%) and employed (83%). About 72% of the women were in medium socioeconomic status category (measured based on subjective perception of the women about sufficiency of the financial resources) and 11% indicated low socioeconomic status. The mean maternal preprepregnancy BMI was 22 kg/m^2^ (±3.6 kg/m^2^). About 9% of the women indicated alcohol consumption during pregnancy.

### 3.2. Characteristics of the Exposure and Outcome Variables

The characteristics of exposure and outcome variables are presented in Table 2. The geometric mean cord BLL was 1 *μ*g/dL with the range 0.4–5.7 *μ*g/dL, and the geometric mean hair mercury level was 0.2 *μ*g/g (range 0.02–1.5 *μ*g/g). Based on cotinine level in saliva, 15% of women were identified as smokers (cotinine level > 10 ng/mL) at the beginning of pregnancy and 12% in third trimester of pregnancy. The mean cotinine level in child urine was 7.1 ng/mL (range < LOD–66.7 ng/mL) for one-year-old and 8.1 ng/mL (range < LOD–32.3 ng/mL) for two-year-old children. The range of 1-HP in urine of pregnant women was from 0.01 to 8.5 *μ*g/g creatinine with geometric mean 0.4 *μ*g/g creatinine. 

The mean composite scores for cognitive, language, and motor development were on average or high average level at one- and two-year-old assessments. The mean composite score for all of the scales was higher than 103 at 12 months and higher than 101 for 24 months. 

### 3.3. Impact of Exposure to Environmental Factors on Child Neurodevelopment

Table 3 presents the adjusted effects of prenatal (for lead, mercury, ETS, and PAH) and postnatal (for ETS) exposures on cognitive, language, and motor abilities. There were no statistically significant effects of prenatal exposure to mercury and 1-HP on child psychomotor development based on linear regression estimation with random effect together for children surveyed in the first and second year of life (*P* ≤ 0.2). For those exposures there were no justification for separate models for one- and two-year-old groups (*P* for interaction between age and biomarker of exposure ≥ 0.4). Combined analysis for both age groups did not indicated significant impact of prenatal lead exposure on child cognitive development (*β* = −2.2; *P* = 0.1). Separate analysis indicated no adverse effect of prenatal lead exposure on cognitive score at 12 months of age (*β* = −0.3; *P* = 0.8) but subsequent testing of children at 24 months of age showed the significant inverse association between lead exposure and cognitive function (*β* = −6.7; *P* = 0.04). The significant negative impact of prenatal lead exposure was observed for language abilities (*β* = −3.2; *P* = 0.04) but not for motor function (*β* = −2.3; *P* = 0.2). Both prenatal and postnatal ETS exposure had significant adverse effects on cognitive (prenatal exposure: *β* = −0.8; *P* = 0.01; postnatal exposure: *β* = −0.2; *P* < 0.001) and language function (prenatal exposure: *β* = −0.9; *P* < 0.001; postnatal exposure: *β* = −0.2; *P* < 0.001). There was no justification for separate analysis at one- and two-year-of age (*P* for interaction between age and biomarker of exposure ≥ 0.2). The significant deficit in motor abilities due to prenatal and postnatal ETS exposure has been observed at 24 months (prenatal exposure: *β* = −1.9; *P* < 0.001; postnatal exposure: *β* = −0.7; *P* < 0.001) but not at one year of age (prenatal exposure: *β* = −0.6; *P* = 0.1; postnatal exposure: *β* = −0.1; *P* = 0.1). 

The model with the adjustment for additional confounders (parental age, parental education, child gender, and for cognitive development additionally maternal marital status and child nursery attendance) indicated an adverse effect of prenatal exposure to ETS on motor development at 24 months (*β* = −1.8; *P* = 0.02) and postnatal ETS exposure on cognitive and language abilities (*β* = −0.2; *P* = 0.02) at all age assessments and motor abilities at 2 years of age (*β* = −0.5; *P* = 0.02). After additional adjustment for cotinine level during first years of life, prenatal exposure to tobacco constituents significantly affects child motor abilities at age of two (*β* = −2.6; *P* = 0.02) (Table 4). Additionally if cotinine level during pregnancy is included as additional confounder, the negative impact of postnatal exposure to tobacco constituents on child psychomotor development remains significant for cognitive (*β* = −0.2; *P* = 0.05) and motor functions (*β* = −0.5; *P* = 0.01). 

The adverse effect of prenatal lead exposure on cognitive score at 24 months was of the boarder significance (*β* = −6.2; *P* = 0.06) after adjustment examiner, parental age and education, marital status, child gender, and child nursery attendance (Table 3). If cotinine levels during pregnancy and within first years of life were added into the analysis the impact of lead exposure on child cognitive abilities at 2 years of age was not statistically significant (*β* = −9.4; *P* = 0.1) (Table 4).

## 4. Discussion

The current study found no statistically significant effects of prenatal exposure to mercury or 1-HP, within the ranges measured, on child psychomotor development. However, we found adverse effects of prenatal exposure to ETS on motor development at 24 months, and for postnatal ETS exposure on cognitive and language abilities at all age assessments as well as motor abilities at 2 years of age. When cotinine level during pregnancy was included as additional confounder, the negative impact of postnatal exposure to tobacco constituents on child psychomotor development remained significant for cognitive and motor functions. The adverse effect of prenatal lead exposure on cognitive score at 24 months was of borderline significance. 

The existing evidence for adverse effects of prenatal mercury exposure on children neurodevelopment is inconclusive. An analysis performed on 233 1-year-old children from Krakow showed an increased risk for delayed performance when cord blood mercury level was greater than 0.80 *μ*g/L [8]. An analysis performed by Jedrychowski et al. on a larger group of children (374) with assessment at one, two, and three years of age [9] indicated an inverse association between mercury exposure and both mental and psychomotor scores only at 12 months of age. No association between exposure to mercury and child neurodevelopment was noticed in other studies [10–14]. The explanation for the inconclusive finding could be different exposure assessment (blood mercury level or hair mercury level) or it can result from the fact that fish and other seafood may contain beneficial nutrients such as n-3 fatty acids as well as harmful contaminants such as mercury. The lack of consistency in the results as well as the presence of possibly modifying factors should be studied more deeply. 

The published studies that have estimated the impact of environmental PAH exposure on child psychomotor development derived from US, Chinese, and Polish cohorts. Most of the analyses show statistically significant associations between the exposure of interest, alone or in combination with ETS exposure, and child psychomotor abilities. In the study performed in Krakow, higher (>17.96 ng/m^3^) prenatal exposure to airborne PAH was associated with a significant reduction in scores on a test of nonverbal intelligence in 5-year-old children. The relationship remained significant after controlling for postnatal exposure to PAH and ETS in the home, maternal intelligence, and lead [18]. In our analysis, we have chosen 1-HP as biomarker of exposure, whereas other researchers used a series of PAH metabolites and performed exposure assessment based on personal air sampling or measurements of PAH-DNA adducts levels. The analysis based on other biomarkers of PAH exposure could be the advantage of other studies performed in the future based on REPRO_PL cohort. 

Our findings support recent studies that BLL much below 10 ug/dL, as recommended by the CDC and WHO, can be associated with impaired cognitive development. A series of analysis about the impact of lead exposure on child mental development have been performed previously based on Krakow cohort [28–30]. Such analyses performed among 6-month-old infants, whose mothers were exposed to low but varying amounts of lead during pregnancy, indicated that the estimated risk of developmental delay was two-fold greater for high BLL compared to low BLL (OR = 2.3; 95% CI 1.3–4.1) [28]. As indicated by Jedrychowski et al. an adverse effect of prenatal lead exposure on mental development was of borderline significance for the 12 months assessment (*β* = −5.4, 95% CI −11.2 to 0.4) and statistically significant for subsequent testing at 24 and 36 months of age (*β* = −7.7, 95% CI −14.7 to −0.6 and *β* = −6.7, 95% CI −12.5 to −0.9, resp.) [30]. In our analysis, the effects of lead exposure on child cognitive development were observed only at older age which can have several explanations. Firstly, the tools selected for child psychomotor evaluation may not be sensitive enough for small children. Secondly, the lack of association for the youngest children can result from difficulties in performing the test among small kids. Additionally, in current study we were able to assess only prenatal lead exposure (based on cord BLL), but it is important to notice that postnatal exposure can be also associated with decreased child mental development. Taking this into account, it needs to be stressed that the effect of prenatal lead exposure can be strengthen by a postnatal one. This can be especially addressed to the children around their second year of life as the consequence of their walking and playing activity (especially outdoors) and touching or placing their hand in their mouth. It is reasonable to establish a longer followup to be able to estimate effects after the first 24 months. The results of the presented analysis and other studies show a need for redefining the primary prevention standards of childhood lead exposure.

The effect of maternal active and passive smoking during pregnancy and ETS exposure after birth on neurodevelopment in young children (age 4 or younger) has been inconsistent. The mostly pointed limitation of many published studies is the retrospective assessment of exposure level and the lack of adequate control for confounding factors. Our preliminary data on REPRO_PL cohort performed on smaller sample size (63 children) indicated a statistically significant association between prenatal exposure to ETS and cognitive child development (*β* = −4.0; *P* = 0.04) and no statistically significant association with motor and language abilities [31]. The current analysis is much improved compared to the previous one. Firstly, it is performed with a larger sample size, which increases the power of the study. Secondly, it focused on data from both one- and two-year-old children (the previous analysis was performed only for one-year-old children). Additionally, in the current analysis we have performed better estimation of smoking status and ETS exposure during pregnancy, taking into account any changes in exposure level throughout the pregnancy period, and we have assessed children's ETS exposure after birth based on cotinine level in urine (not on questionnaire data as it was done previously). Finally, the current analysis allows for the inclusion of a variety of confounding factors. 

### 4.1. Study Design

The current analysis is based on the Polish Mother and Child Cohort Study (REPRO_PL cohort). The prospective study design with well-assessed exposure levels based on biomarker measurements is the advantage of the current analysis. The study design also enables notification of any changes in exposure level which is crucial for valid assessment of tobacco smoking. Additionally, a series of detailed questionnaires (collected once in each trimester of pregnancy, one week and one/two years after delivery) allow for reliable assessment of exposure and confounding variables. The limitation of the current study is the relatively small sample size, especially for multivariable analysis at the age of 24 months with the inclusion of few exposures (lead and pre/postnatal tobacco) and variety of confounders. 

### 4.2. Exposure Assessment

Valid exposure assessment based on biomarkers measurement is a crucial part and the main advantage of the current study. For the assessment of lead exposure, cord blood level was selected, which is frequently used (together with maternal BLL during pregnancy) in the measurement of prenatal lead exposure [28–30, 32]. For exposure assessment after birth, a series of measures of BLL in children are chosen by various researchers [3, 33–36] which were not performed in the current study and established its main limitation. The blood sample was not collected from children because of following reasons: difficulties in performing blood sample collection and lack of parental interest and agreement for blood sampling among such young children. Although we have observed borderline adverse effect of prenatal lead exposure on cognitive score, the impact of postnatal exposure cannot be excluded. In the current study, the cord BLL was low (GM = 1.0 *μ*g/dL; GSD ± 0.7) with only one sample that slightly exceeded 5 *μ*g/dL. The majority of studies in this field also looked at BLL below 10 *μ*g/dL. The analysis based on the Krakow cohort showed a similar cord BLL as observed in our REPRO_PL cohort (median cord BLL 1.2 *μ*g/dL and range 0.4–6.9 *μ*g/dL) [30].

The level of mercury in the umbilical cord and maternal blood, as well as in maternal or fetal hair [4, 5, 7–9, 12, 13], is frequently used for assessment of exposure to this metal. Hair strands are the best indicator of past mercury exposure [11]. In our study the mean hair mercury level was 0.2 *μ*g/g (range 0.02–1.5 *μ*g/g). The levels found in other studies range from 0.6 ppm [4] for a US cohort to 6.8 ppm [14] for cohort from the Republic of Seychelles. The level of mercury is highly determined by fish consumption. Based on REPRO_PL analysis (unpublished data), the women consumed fish at a low or moderate level (about 25% of the women indicate fish consumption less frequently than once per week). 

In published studies the prenatal PAH exposure assessment is mostly based on personal air sampling during pregnancy [15, 17, 18] and PAH-DNA adducts measured in cord blood [16, 19–21]. In our current study, 1-HP level in urine collected during the second trimester of pregnancy was selected as the biomarker of PAH exposure. In the studies performed by Perera et al. [15, 17] and Edwards et al. [18] the authors considered a single monitoring time point to be a reasonable indicator of prenatal exposure via inhalation over the last two trimesters of pregnancy because measurements during the second and third trimesters were correlated. This is proved by our previous analysis based on REPRO_PL cohort in which we have not observed statistically significant differences in 1-HP level between measurements in the second and third trimester of pregnancy (*P* = 0.7) [37]. In the current analysis, the range of 1-HP in urine of pregnant women was from 0.01 to 8.5 *μ*g/g creatinine with a geometric mean 0.4 *μ*g/g creatinine. In other analysis performed in Poland the mean concentration of 1-HP in urine among smokers was 0.2 *μ*mol/mol creatinine and among nonsmokers 0.1 *μ*mol/mol creatinine [38]. It would be reasonable to assess also other metabolites of PAH but, unfortunately, that was not feasible in this analysis. 

Most of the studies evaluating the impact of ETS exposure on child neurodevelopment have measured the exposure based on questionnaire data [22]. Some of them collect data prospectively (the birth cohort studies), but there are also studies which use retrospective report of smoking during pregnancy. Data collected prospectively gives better estimation of exposure level. Only few of the studies have verified the exposure level during pregnancy by biomarker level. As far as we know, this analysis is the first with well-assessed pre- and postnatal exposure levels to tobacco constituents. The prospective study design, detailed questionnaire data, and biomarker measurements (cotinine level in saliva assessed 3 times in pregnancy and cotinine level in child urine assessed at 12 and 24 months) allowed for valid assessment of exposure and opened for changes in exposure level (such as changes in smoking status during pregnancy). In our analysis, 15% of women were identified as smokers (cotinine level > 10 ng/mL) in the first trimester and 12% in the third trimester of pregnancy. These results are in agreement with data from a survey conducted in Poland on 2654 pregnant women which found that 25% of women smoked 3 months prior to conception, and that 12% continue with smoking during pregnancy [39]. Based on the existing knowledge both pre- and postnatal ETS exposure are the risk factors for poorer child psychomotor development. Taking into account that mothers who smoke during pregnancy continue smoking after child birth, it is difficult to judge which exposure is more dangerous for child neurodevelopment. 

### 4.3. Outcome Variables

In order to capture the early cognitive, motor, and language outcomes of environmental exposures, we have chosen the Bayley Scales of Infant and Toddler Development (third edition) the revision of the Bayley Scales of Infant Development (second edition). This is a well standardized and the most common tool used for evaluating neurodevelopmental effects in young children [8, 9, 13, 15, 16, 19, 22, 29, 30]. The tests offer early and fairly comprehensive measures of child development. It is pointed out that the tests can be more variable and not sensitive in younger children. Our study indicated a significant effect of prenatal lead exposure on cognitive development as well as effect of pre/postnatal ETS exposure on motor abilities for children tested at age of 24 months but not at younger age. Other studies evaluating the impact of environmental exposures on child neurodevelopment have also observed such effects at older but not younger ages. This may be due to difficulties in testing very young children or due to the fact that effect of pollutants may become apparent only with maturation over time. In the current study, the child psychomotor testing was done by 4 trained psychologists or child developmental specialists using the same tools. To eliminate the impact of examiner all analysis were performed with the adjustment of the person who performed the test. The child neurodevelopment examination was performed in the presence of the mother or relative to guarantee sense of safety and increase child willingness to cooperate with examiner. 

### 4.4. Confounders

We assessed the potential for confounding for a wide range of data on socioeconomic factors and lifestyle habits (parental age, parental education, marital status, socioeconomic status, alcohol consumption during pregnancy, child gender, type of delivery, gestational age, biometric indicators at birth, breastfeeding, number of siblings, and nursery attendance). Still we cannot exclude the possibility that confounding by unmeasured risk factors (e.g., maternal IQ, children's maternal relationship, and home environment) produced associations between environmental exposures and child neurodevelopment. 

## 5. Conclusions

The study found a negative impact of prenatal and postnatal exposure to environmental tobacco smoke on child psychomotor development within first two years of life. Moreover, an adverse effect of prenatal exposure to lead was of borderline significance. These results underscore the importance of policies and public health interventions that reduce such exposures. To determine whether the psychomotor deficit documented in this study persists to older ages, the followup of the children over the next several years will be carried out. 

## Figures and Tables

**Table 1:** Child and parental characteristics.

Variables	*n* or mean	% or SD
Gender of the child (*N* = 406)		
Boys	192	47.3
Girls	214	52.7
Birth weight (g); mean, SD (*N* = 388)	3352.5	488.3
Gestational age (weeks); mean; SD (*N* = 370)	39.3	1.4
Length (cm); mean, SD (*N* = 388)	54.9	2.9
Head circumference (cm); mean, SD (*N* = 384)	34.3	1.6
Chest circumference (cm); mean, SD (*N* = 382)	33.4	1.8
Breastfeeding (months) (*N* = 406)		
No	66	16.3
<6	156	38.4
≥6	184	45.3
Nursery attendance at 12 months (*N* = 406)		
Yes	27	6.7
No	379	93.3
Nursery attendance at 24 months (*N* = 193)		
Yes	36	18.7
No	157	81.3
Age at Bayley test administration (months)		
For one year old; mean, SD (*N* = 406)	12.4	1.3
For two years old; mean, SD (*N* = 198)	24.4	1.7
Number of siblings (*N* = 406)		
0	234	57.6
≥1	172	42.4
Maternal age (years); mean, SD (*N* = 406)	29.8	4.4
Paternal age (years); mean, SD (*N* = 398)	31.8	5.5
Maternal education (*N* = 406)		
Primary/vocational	33	8.1
Secondary	134	33.0
University	239	58.9
Paternal education (*N* = 401)		
Primary/vocational	79	19.7
Secondary	161	40.1
University	161	40.1
Marital status (*N* = 406)		
Married	311	76.6
Unmarried	95	23.4
Maternal employment (*N* = 406)		
Employed	338	83.3
Unemployed	68	16.7
Socioeconomic status (*N* = 406)		
High	71	17.5
Medium	292	71.9
Low	43	10.6
Maternal prepregnancy BMI (kg/m^2^); mean, SD (*N* = 406)	22.3	3.6
Type of delivery (*N* = 366)		
Cesarean	129	35.2
Vaginal	237	64.8
Alcohol consumption during pregnancy (*N* = 259)		
Yes	22	8.5
No	237	91.5

**Table 2:** Characteristics of the exposure and outcome variables.

Variables	Geometric mean	Mean	SD	Median	Min	Max
Cord blood lead level (*μ*g/dL); *N* = 233	1.0	1.1	0.7	1.0	0.4	5.7
Hair mercury (*µ*g/g); *N* = 227	0.2	0.3	0.2	0.2	0.02	1.5
Cotinine level in saliva (ng/mL)						
1st trimester of pregnancy; *N* = 362	2.1	19.1	54.7	1.4	<LOD	400
2nd trimester of pregnancy; *N* = 132	2.2	26.1	64.2	2.3	<LOD	376
3rd trimester of pregnancy; *N* = 345	1.8	14.3	43.3	1.3	<LOD	339.5
Cotinine level in child urine (ng/mL)						
For one-year-old children; *N* = 228	3.6	7.1	9.5	3.7	<LOD	66.7
For two-year-old children; *N* = 116	5.3	8.1	7.0	5.8	<LOD	32.3
1-Hydroxypyrene level in urine of pregnant women (*μ*g/g creatinine); *N* = 384	0.4	0.5	0.6	0.4	0.01	8.5
Composite score for one-year-old children; *N* = 406						
Cognitive		106.0	10.8	105.0	80.0	145.0
Language		107.6	13.5	109.0	72.0	141.0
Motor		103.9	13.4	103.0	73.0	142.0
Composite score for two-year-old children; *N* = 198						
Cognitive		109.0	15.7	105.0	80.0	145.0
Language		101.3	13.3	97.0	74.0	144.0
Motor		112.6	15.5	110.0	73.0	154.0

LOD: limit of detection.

**Table 3:** Association between exposure to environmental factors and child cognitive, language, and motor development.

Biomarkers of exposure	Model 1			Model 2		
	*β* (95% CI)			*β* (95% CI)		
	Cognitive	Language	Motor	Cognitive	Language	Motor
*Cord blood lead level (log transformed) *						
Psychomotor score at 12 and 24 months combined (*n* = 326)	−2.2 (−5.0 to 0.7)*	−3.2 (−6.2 to −0.2)	−2.3 (−5.6 to 0.9)		−2.6 (−5.6 to 0.4)^e^	
Psychomotor score at 12 months (*n* = 233)	−0.3 (−3.1 to 2.5)					
Psychomotor score at 24 months (*n* = 93)	−6.7 (−13.3 to −0.1)			−6.2 (−12.8 to 0.5)^b^		
*Hair mercury *						
Psychomotor score at 12 and 24 months combined (*n* = 303)	1.6 (−4.4 to 7.6)	2.7 (−3.9 to 9.4)	5.3 (−2.2 to 12.9)			
*Saliva cotinine level during pregnancy* ^ a^ *(log transformed) *						
Psychomotor score at 12 and 24 months combined (*n* = 590)	−0.8 (−1.4 to −0.2)	−0.9 (−1.6 to −0.3)	−0.9 (−1.6 to −0.3)*	−0.3 (−1.0 to 0.4)^c^	−0.5 (−1.2 to 0.2)^c^	−0.9 (−1.6 to −0.1)^c^
Psychomotor score at 12 months (*n* = 397)			−0.6 (−1.3 to 0.2)			
Psychomotor score at 24 months (*n* = 193)			−1.9 (−3.2 to −0.7)			−1.8 (−3.3 to −0.3)^f^
*Cotinine level in child urine *						
Psychomotor score at 12 and 24 months combined (*n* = 333)	−0.2 (−0.4 to −0.1)	−0.2 (−0.4 to −0.1)	−0.2 (−0.4 to −0.1)*	−0.2 (−0.3 to −0.03)^d^	−0.2 (−0.3 to −0.03)^d^	−0.2 (−0.3 to 0.01)^d^
Psychomotor score at 12 months (*n* = 228)			−0.1 (−0.3 to 0.03)			
Psychomotor score at 24 months (*n* = 105)			−0.7 (−1.1 to −0.3)			−0.5 (−0.9 to −0.1)^g^
*1-Hydroxypyrene level in pregnant women urine *						
Psychomotor score at 12 and 24 months combined (*n* = 576)	−0.7 (−2.3 to 1.0)	−0.8 (−2.5 to 0.9)	−1.3 (−3.1 to 0.6)			

*Interaction between age and biomarker of exposure *P* ≤ 0.05.

^a^Cumulative value for prenatal period.

^b^ *N* = 92; ^c^ *N* = 576; ^d^ *N* = 325; ^e^ *N* = 319; ^f^ *N* = 189; and ^g^ *N* = 104.

Model 1—adjusted for examiner and age at assessment (one or two years of age).

Model 2—adjusted for examiner, age at assessment for combined analysis (one or two years of age), parental age, parental education, child gender, and for cognitive development additionally marital status and child nursery attendance.

**Table 4:** Association between exposure to lead and tobacco constituents and child cognitive, language, and motor-development-multivariate model.

Biomarkers of exposure	Model 3		
	*β* (95% CI)		
	Cognitive	Language	Motor
Cord blood lead level (log transformed)^a^			
Psychomotor score at 24 months (*N* = 43)	−9.4 (−21.3 to 2.4)		
Cotinine level during pregnancy (log transformed)			
Psychomotor score at 24 months (*N* = 100)^b^			−2.6 (−4.7 to −0.4)
Cotinine level in child urine^c^			
Psychomotor score at 12 and 24 months combined (*n* = 317)	−0.2 (−0.3 to −0.001)	−0.2 (−0.3 to 0.02)	
Psychomotor score at 24 months (*N* = 100)			−0.5 (−0.9 to −0.2)
Cotinine level in child urine^d^			
Psychomotor score at 12 and 24 months combined (*n* = 159)	−0.2 (−0.5 to 0.06)		

^a^Adjusted for examiner, parental age, parental education, child gender, marital status, child nursery attendance, cotinine level during pregnancy, and within first years of life.

^b^Adjusted for examiner, parental age, parental education, child gender, and cotinine level within first years of life.

^c^Adjusted for examiner, age at assessment for combined analysis (one or two years of age), parental age, parental education, child gender, cotinine level during pregnancy, and for cognitive development additionally marital status and child nursery attendance.

^d^Adjusted for examiner, age at assessment (one or two years of age), parental age, parental education, child gender, marital status, child nursery attendance, cotinine level during pregnancy, and cord blood lead level.
